# Effects of High-Monounsaturated-Fatty-Acid (MUFA) Diet and Melatonin Supplementation on Lipid Metabolism in Female Rats

**DOI:** 10.3390/biology15060515

**Published:** 2026-03-23

**Authors:** Jun-Ling Luo, Yi-Wen Chien

**Affiliations:** 1 Department of Nutrition and Health Science, Taipei Medical University, Taipei 11031, Taiwan; 2 Research Center of Geriatric Nutrition, College of Nutrition, Taipei Medical University, Taipei 11031, Taiwan; 3 Graduate Institute of Metabolism and Obesity Sciences, Taipei Medical University, Taipei 11031, Taiwan; 4 Nutrition Research Center, Taipei Medical University Hospital, Taipei 11031, Taiwan; 5 TMU Research Center for Digestive Medicine, Taipei Medical University, Taipei 11031, Taiwan

**Keywords:** fatty acid ratio, melatonin, lipid metabolism, gene expression, female rats

## Abstract

Background: This study investigated the effects of melatonin (50 mg/kg/day) and a high-monounsaturated-fatty-acid (MUFA) diet (60% MUFAs; PUFA/SFA ratio = 5), alone or combined, on lipid metabolism-related gene expression in female Sprague–Dawley rats. Methods: Thirty-two eight-week-old rats were randomized into four groups: control (C), melatonin (M), experimental oil diet (E), and combined melatonin plus experimental oil diet (ME). Treatments lasted eight weeks, with melatonin given orally at 17:00–19:00 daily. Results: In the ME group, hepatic expression of peroxisome proliferator-activated receptor α (PPARα) and acyl-CoA oxidase (ACO) mRNA was markedly upregulated, while the E group showed significantly higher expression of hormone-sensitive lipase (HSL) in white adipose tissue. Additionally, ME rats exhibited increased fibronectin type III domain-containing protein 5 (FNDC5) mRNA with reduced peroxisome proliferator-activated receptor gamma coactivator-1α (PGC-1α), but serum irisin remained unchanged. Conclusions: Melatonin and a high-MUFA diet induced modest and non-uniform changes in lipid metabolism-related gene expression. However, these transcriptional responses were not accompanied by measurable physiological or metabolic outcomes, indicating that the observed effects represent early molecular adaptations rather than functional metabolic modulation.

## Simple Summary

This study explored whether melatonin supplementation and a high-monounsaturated-fatty-acid (MUFA) diet were associated with changes in lipid metabolism-related gene expression in female rats. After eight weeks, no significant changes were observed in body weight or blood lipid profiles. However, the combination of melatonin and the high-MUFA diet enhanced the expression of hepatic genes involved in fat oxidation, including peroxisome proliferator-activated receptor α (PPARα) and acyl-CoA oxidase (ACO), and the high-MUFA diet increased hormone-sensitive lipase (HSL) expression in adipose tissue. Interestingly, this combined treatment also upregulated fibronectin type III domain-containing protein 5 (FNDC5) while downregulating peroxisome proliferator-activated receptor gamma coactivator-1α (PGC-1α). These findings indicate that melatonin and dietary fatty acid composition are associated with distinct molecular responses related to lipid metabolism; however, these responses did not translate into clear physiological benefits, highlighting the need for longer-term studies to clarify their biological significance.

## 1. Introduction

The occurrence of obesity and related metabolic conditions in women of childbearing age has been on a steady incline in recent times [1]. These conditions not only impair reproductive health but also elevate future risks of cardiovascular disease, insulin resistance, and postmenopausal metabolic syndrome [2,3]. Estrogen plays a key role in regulating energy homeostasis and lipid metabolism, making the reproductive-age period—characterized by hormonal fluctuations—an important window for interventions aimed at preventing fat accumulation and metabolic imbalances. Estradiol, in particular, not only regulates female reproductive functions but also influences energy intake, lipid metabolism, and the body fat distribution [4,5]. Shifts in estradiol concentration are significantly correlated with obesity and impaired metabolic regulation [4,6]. This phenomenon is particularly pronounced in menopausal women, as the reduction in estrogen levels is associated with greater fat accumulation and an elevated risk of developing cardiovascular diseases.

As reported previously, melatonin, secreted by the pineal gland, regulates circadian rhythm and energy balance [7]. Beyond its role as the “sleep hormone,” it exerts antioxidant, anti-inflammatory, and anti-apoptotic effects [8]. Melatonin activates the 5′ adenosine monophosphate-activated protein kinase (AMPK) pathway, suppressing lipogenic enzymes such as acetyl-CoA carboxylase (ACC) and sterol regulatory element-binding protein-1c (SREBP-1c), while enhancing fatty acid oxidation genes including carnitine palmitoyltransferase-1 (CPT-1) and peroxisome proliferator-activated receptor gamma coactivator-1α (PGC-1α), thereby reducing lipid synthesis and promoting mitochondrial activity [9,10,11]. In male Sprague–Dawley (SD) rats, oral melatonin (10 mg/kg/day, 60 days) reduced serum triglycerides (TGs), high-density lipoprotein cholesterol (HDL-C), low-density lipoprotein cholesterol (LDL-C), and total cholesterol (TC), accompanied by decreased fatty acid synthase (FAS) and increased CPT-1 expression [12]. Similarly, in diet-induced dyslipidemia hamsters, melatonin (10–50 mg/kg/day, 8 weeks) lowered hepatic TGs and TC by downregulating lipogenic pathways [13].

Beyond hormonal influences, the proportion of dietary fatty acids (FAs) is also a critical determinant in regulating lipid metabolism and associated inflammatory processes [14]. Recent research has increasingly focused on not only total fat intake but also the specific proportions of dietary fatty acids [15]. A study in male Wistar rats demonstrated that increasing the proportion of monounsaturated fatty acids (MUFAs), compared to a saturated fatty acids (SFAs)-rich-diet, improved insulin sensitivity, enhanced adiponectin activity, and reduced LDL-C levels [16]. Our previous research also indicated that the dietary FA composition exerts differential effects on fat accumulation in both normal-weight and obese rodents. Under high-fat dietary conditions, modifying the FA profile—specifically by increasing the MUFA content and raising the ratio of PUFA to SFA (P/S ratio)—can enhance the activity of lipid catabolic enzymes, thereby effectively reducing BW gain and adiposity [17,18].

Therefore, the present study was designed to characterize the transcriptional responses of lipid metabolism-related genes to a defined high-MUFA dietary fatty acid composition and melatonin supplementation in female rats under non-obese, physiologically normal conditions. Rather than evaluating obesity prevention or therapeutic efficacy, this study aimed to examine whether these interventions elicit early molecular adaptations in hepatic and adipose tissues in the absence of overt metabolic dysfunction. To this end, female rats received daily oral administration of melatonin (50 mg/kg BW) in combination with a high-MUFA diet (MUFAs = 60%, PUFA/SFA ratio = 5) for an 8-week period, and changes in serum lipid profiles and lipid metabolism-related gene expression were assessed.

## 2. Materials and Methods

### 2.1. Animals

The study utilized 32 female Sprague–Dawley rats, eight weeks old, obtained from BioLasco (Taipei, Taiwan). They were housed in a controlled setting with a 12:12 h light/dark cycle (lights activated at 07:00), ambient temperature maintained at 22 ± 2 °C, and relative humidity at 65 ± 5%. Following a 1-week acclimatization phase, rats were provided free access to Rodent Laboratory Chow 5001 (PMI Nutrition International, St. Louis, MO, USA) and water. The study protocol was approved by the IACUC of Taipei Medical University (LAC-2022-0370).

### 2.2. Experimental Oil Diet Composition

The experimental diet was formulated based on a modified AIN-93M composition, following a previous study from our laboratory [19], with the ingredient composition shown in Table 1. Each week, the powdered diet was mixed in a feed blender, and a lipid mixture consisting of 93% canola oil and 7% soybean oil (both from Uni-President Enterprises, Taiwan) was added and thoroughly blended. The prepared diet was then cut into portions and stored at 4 °C.

### 2.3. Experimental Design

Thirty-two rats were randomly divided into four experimental groups (*n* = 8 per group) after one week of acclimatization. Baseline BW values were not significantly different among the groups prior to intervention, according to statistical analysis. The groups included: a control group (C), which received an 8% (*w*/*v*) ethanol vehicle and a 40% fat diet based on soy-bean oil; a melatonin group (M), which received melatonin at 50 mg/kg BW/day along with a 40% soybean oil diet; an experimental oil group (E), which was administered 1 mL/kg BW/day of 8% (*w*/*v*) ethanol and a 40% fat diet containing a specially formulated oil mixture with a P/S = 5 and 60% MUFAs, but no melatonin; and a melatonin plus experimental oil (ME) group, which received 50 mg/kg BW/day melatonin combined with the same oil-based diet as the E group (P/S = 5, MUFAs = 60%). Melatonin (Sigma-Aldrich, St. Louis, MO, USA) solutions were prepared fresh daily in 8% (*w*/*v*) ethanol, protected from light during preparation, and kept at 4 °C in darkness. The melatonin dose was set at 50 mg/kg BW/day, based on the results of our previous experiments, where this dosage was shown to improve lipid metabolism in ovariectomized (OVX) rats [20]. All interventions were administered by oral gavage between 17:00 and 19:00, approximately 1–2 h before lights-off. Throughout the 8-week intervention, BW and dietary intake were recorded on a weekly basis. The food efficiency ratio (FER, %) was calculated as the ratio of BW gain to food intake. At the end of the study, the rats were euthanized using an intraperitoneal injection of a Zoletil (Virbac, Carros, France)–Rompun (Bayer, Leverkusen, Germany) combination anesthetic at a dosage of 1 mL/kg BW. Blood was drawn and centrifuged at 3500× *g* for 15 min at 4 °C to obtain serum. The uterus, liver, gonadal white adipose tissues (gWATs), perirenal WATs (pWATs), quadriceps muscles, and gastrocnemius muscles were weighed. All collected serum and tissue samples were kept at −80 °C prior to subsequent examinations.

### 2.4. Serum Measurements

Serum follicle-stimulating hormone (FSH) and estradiol levels were quantified using enzyme-linked immunosorbent assay (ELISA) kits supplied by Wuhan Fine Biotech (Wuhan, China). The serum TC, TGs, HDL-C, and free FAs (FFAs) were analyzed by colorimetric assay kits (Randox Laboratories, Crumlin, UK). Serum LDL-C levels were calculated using the formula LDL-C (mg/dL) = TC (mg/dL) − HDL-C (mg/dL) − TGs (mg/dL)/5, and are reported as estimated values, which should be interpreted with caution in rodents [21,22]. Leptin levels were assessed via ELISA (BioVendor, Czech Republic), while adiponectin and irisin were measured using respective kits from AssayPro (St. Charles, MO, USA) and BioVendor (Brno, Czech Republic).

### 2.5. Hepatic Lipid Measurements

Liver lipid profiles were obtained following the Folch extraction procedure [23], and the concentrations of TC, TGs, and FFA (mg/g liver tissue) were determined using colorimetric assay kits from Randox Laboratories (Crumlin, UK).

### 2.6. Real-Time Quantitative Polymerase Chain Reaction (RT-qPCR)

Total RNA was isolated from the liver, gWATs, and quadriceps muscle using Trizol reagent (Life Technologies, Carlsbad, CA, USA) according to the manufacturer’s instructions [20]. The mRNA expression of lipid metabolism-related genes, including ACC, FAS, SREBP-1c, AMPK, CPT-1, PGC-1α, acyl-CoA oxidase (ACO), lipoprotein lipase (LPL), hormone-sensitive lipase (HSL), peroxisome proliferator-activated receptor α (PPARα), peroxisome proliferator-activated receptor γ (PPARγ), and fibronectin type III domain-containing protein 5 (FNDC5), was analyzed by RT-qPCR as previously described [18]. β-actin served as the internal control, and relative expression levels were calculated using the 2^−ΔΔCt^ method. Primer sequences are provided in Table 2.

### 2.7. Histopathological Examination

Hepatic tissues and gWATs were preserved in 10% neutral-buffered formalin, followed by trimming, paraffin embedding, sectioning, and hematoxylin and eosin (H&E) staining. A board-certified veterinary pathologist, blinded to the group assignments, conducted the histological evaluations. The extent of histopathological changes was evaluated based on the scoring systems described in the methods of Shackelford and colleagues as well as Kleiner and co-workers [24,25].

### 2.8. Statistical Analysis

Data are expressed as mean ± standard error of the mean (SEM) and were analyzed using GraphPad Prism version 10.1.0 (GraphPad Software, San Diego, CA, USA). Differences among groups were assessed via one-way ANOVA, followed by Tukey’s post hoc multiple comparisons test. A *p*-value of < 0.05 was considered statistically significant.

## 3. Results

### 3.1. Reproductive Endocrine Variables

At the end of the 8-week intervention with melatonin and the experimental oil diet in 8-week-old female rats, serum estradiol and FSH levels were measured. As shown in Table 3, no significant differences were detected between groups, suggesting that these reproductive endocrine indicators were not markedly affected by the treatments.

### 3.2. BW and Food Intake

Table 4 summarizes BW and dietary intake following the 8-week treatment with melatonin and the experimental oil diet. No statistically significant variations were detected among groups in baseline BW, final BW, weight gain, or FER. In contrast, total food intake and daily energy consumption were significantly greater in the M group relative to the E group (*p* = 0.048).

### 3.3. Liver, Adipose Tissue, and Muscle Tissue Masses

After the 8-week intervention with melatonin and the experimental oil diet, the liver, visceral fat tissues (gWATs and pWATs), and muscle tissues (quadriceps and gastrocnemius) were collected and weighed. Relative weights were calculated by normalizing the organ and tissue weights to BW, as shown in Table 5. No significant group differences were found in the proportional weights of the liver, visceral adipose depots, or skeletal muscle. Liver weight tended to be lower in the M group than in the E group (*p* = 0.083); however, the difference did not reach statistical significance.

### 3.4. Serum Lipid Profile

**Figure 1:**
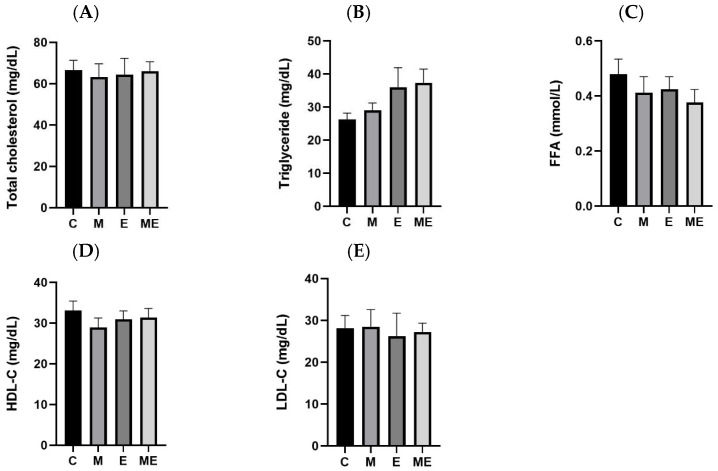
Impact of an 8-week melatonin regimen and experimental oil diet on serum lipid composition in female rats. The figure depicts lipid profile changes following individual or combined treatments with melatonin and the experimental oil diet. (**A**) Serum total cholesterol (TC) levels; (**B**) serum triglycerides (TGs) levels; (**C**) serum free fatty acid (FFA) levels; (**D**) serum high-density lipoprotein cholesterol (HDL-C) levels; (**E**) serum low-density lipoprotein cholesterol (LDL-C) levels. All values are presented as the mean ± standard error of the mean (*n* = 8). No statistically significant differences were identified among the experimental groups using one-way ANOVA with Tukey’s post hoc test. Abbreviations: C, control group; M, melatonin group; E, experimental oil diet; ME, melatonin + experimental oil diet.

Following 8 weeks of melatonin plus experimental oil diet supplementation, serum lipid parameters—TC, TGs, FFAs, HDL-C, and LDL-C—were evaluated. Based on the data in Figure 1, none of these markers differed significantly among the experimental groups.

### 3.5. Serum Adipokines

**Figure 2:**
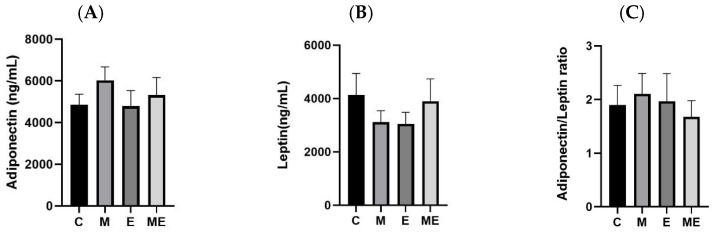
Impact of an 8-week melatonin regimen and experimental oil diet on serum adipokine levels in female rats. The figure illustrates adipokine profiles following individual and combined treatments with melatonin and the experimental oil diet. (**A**) Serum adiponectin levels; (**B**) serum leptin levels; (**C**) serum adiponectin/leptin ratio. All values are presented as the mean ± standard error of the mean (*n* = 8). There were no statistically significant differences identified among the experimental groups using one-way ANOVA with Tukey’s post hoc test. Group abbreviations: C, control; M, melatonin; E, experimental oil diet; ME, melatonin + experimental oil diet.

Serum adipokine levels, including adiponectin and leptin, are presented in Figure 2. Following the 8-week intervention, serum levels of adiponectin, leptin, and their ratio did not differ significantly among the groups.

### 3.6. Hepatic Lipid Profile

**Figure 3:**
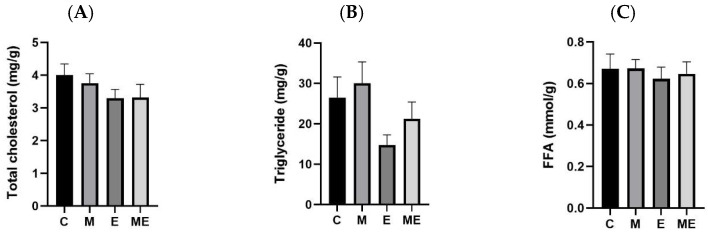
Impact of an 8-week melatonin regimen and the experimental oil diet on hepatic lipid composition in female rats. The figure illustrates changes in liver lipid parameters following combined or individual interventions with melatonin and the experimental oil diet. (**A**) Hepatic total cholesterol (TC) levels; (**B**) hepatic triglycerides (TGs) levels; (**C**) serum free fatty acid (FFA) levels. All values are presented as the mean ± standard error of the mean (*n* = 8). There were no statistically significant differences identified among the experimental groups using one-way ANOVA with Tukey’s post hoc test. Group abbreviations: C, control; M, melatonin; E, experimental oil diet; ME, melatonin + experimental oil diet.

The data in Figure 3 summarize hepatic TC, TG, and FFA levels. At the end of the 8-week melatonin plus experimental oil diet regimen, no significant differences in these lipid measures were observed across groups. A downward shift in TG content was apparent in the E group when contrasted with the M group (*p* = 0.087).

### 3.7. Hepatic mRNA Expression of Lipid Metabolism-Associated Enzymes

**Figure 4:**
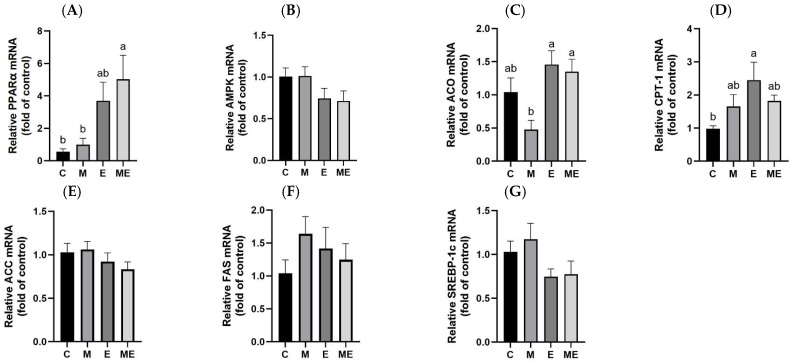
Effects of melatonin supplementation and the experimental oil diet on hepatic fat-metabolism-associated enzyme mRNA levels in female rats. Relative (**A**) peroxisome proliferator-activated receptor alpha (PPAR*α*); (**B**) 5′ adenosine monophosphate-activated protein kinase (AMPK); (**C**) acyl-CoA oxidase (ACO); (**D**) carnitine palmitoyl transferase-1 (CPT-1); (**E**) acetyl-CoA carboxylase (ACC); (**F**) fatty acid synthase (FAS); and (**G**) sterol regulatory element-binding protein 1c (SREBP-1c). Gene expression values were normalized to β-actin using the 2^−ΔΔCt^ approach, with the C group serving as the baseline for relative quantification. Data are expressed as mean ± standard error of the mean based on eight biological replicates per group. *p* < 0.05 was determined statistical significance using one-way ANOVA with post hoc Tukey’s multiple comparisons. Distinct lowercase letters indicate significant differences among the groups. Group abbreviations: C, control; M, melatonin; E, experimental oil diet; ME, melatonin + experimental oil diet.

Figure 4 displays the hepatic expression patterns of genes involved in lipid metabolism after the 8-week treatment with melatonin and the experimental oil-based diet. Figure 4A demonstrates a significant upregulation of PPARα mRNA expression in the ME group relative to both the C (*p* = 0.014) and M groups (*p* = 0.029). In Figure 4C, although ACO mRNA expression did not significantly differ among the groups compared to the C group, it was significantly higher in the E (*p* = 0.005) and ME groups (*p* = 0.015) than in the M group. As shown in Figure 4D, CPT-1 mRNA expression was significantly higher in the E group relative to the C group (*p* = 0.022). In contrast, the expression of AMPK, an upstream regulator of lipid oxidation and breakdown, showed no significant differences among the groups (Figure 4B). The expression of ACC, FAS, and SREBP-1c mRNA, which are key genes in the lipid synthesis pathway, showed no significant intergroup variation.

### 3.8. Hepatic Histopathology

**Figure 5:**
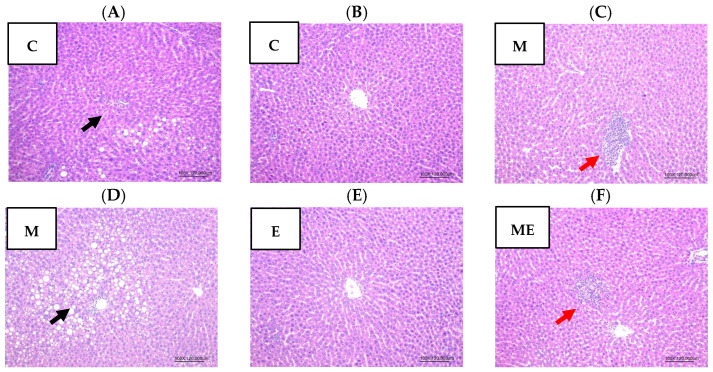
Effects of melatonin supplementation and the experimental oil diet on hepatic histopathology in female rats (100×, H&E). (**A**) Control group (C) showing steatosis (black arrow); (**B**) C group without obvious pathological changes; (**C**) Melatonin group (M) showing inflammatory cell infiltration (red arrow); (**D**) M group showing steatosis (black arrow); (**E**) Experimental oil diet group (E) without obvious pathological changes; (**F**) Melatonin + experimental oil diet group (ME) showing inflammatory cell infiltration (red arrow). Abbreviations: C, control; M, melatonin; E, experimental oil diet; ME, melatonin + experimental oil diet.

After the 8-week intervention with the experimental oil diet and melatonin supplementation, liver tissues from the left lobe were collected and stained with H&E. Histological evaluations under 100× magnification were performed to assess hepatic steatosis, necrosis, and inflammatory cell infiltration, as shown in Figure 5. No hepatic necrosis was observed in any group. Mild to moderate steatosis was noted locally in the C (Figure 5A) and M groups (Figure 5D), while mild focal inflammatory infiltration was observed in the M (Figure 5C) and ME groups (Figure 5F). Although these findings were observed, steatosis, necrosis, and inflammation scores did not vary significantly among the experimental groups.

### 3.9. Adipose Tissue Lipid Metabolism-Related Enzyme mRNA Levels

**Figure 6:**
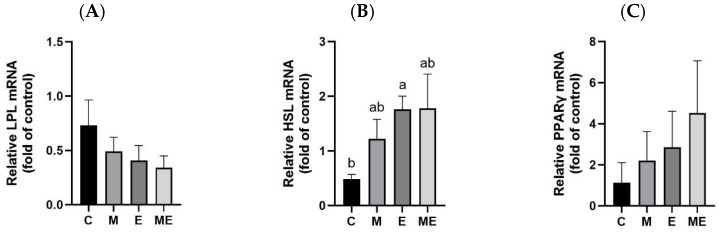
Effects of melatonin supplementation and the experimental oil diet on adipose lipid enzyme mRNA in female rats. Relative (**A**) lipoprotein lipase (LPL); (**B**) hormone-sensitive lipase (HSL); and (**C**) peroxisome proliferator-activated receptor gamma (PPARγ). Gene expression values were normalized to β-actin using the 2^−ΔΔCt^ approach, with the C group serving as the baseline for relative quantification. Data are expressed as mean ± standard error of the mean based on eight biological replicates per group. Statistical significance (*p* < 0.05) was determined using one-way ANOVA with post hoc Tukey’s multiple comparisons. Distinct lowercase letters indicate significant differences among the groups. Group abbreviations: C, control; M, melatonin; E, experimental oil diet; ME, melatonin + experimental oil diet.

Gene expression data for WAT-regulating lipid metabolic pathways, presented in Figure 6, were collected after an 8-week intervention of melatonin with the experimental oil diet. The E group demonstrated a significant rise in HSL mRNA expression versus the C group (*p* = 0.005; Figure 6B). No meaningful differences were observed for LPL or PPARγ among the groups.

### 3.10. White Adipose Tissue (WAT) Morphology

**Figure 7:**
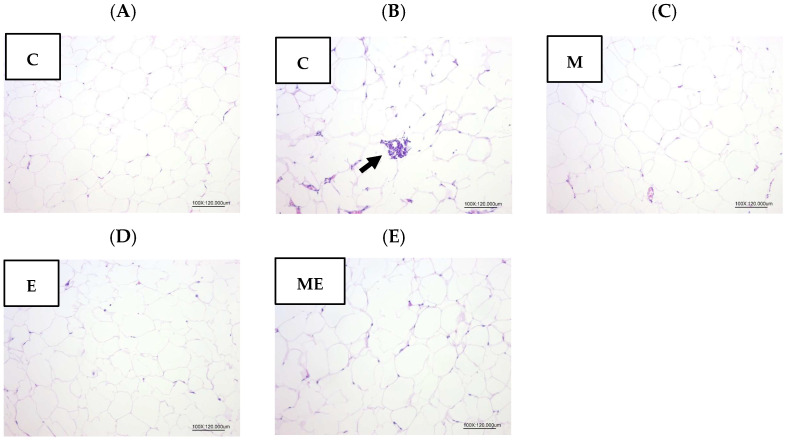
Effects of melatonin supplementation and the experimental oil diet on white adipose tissue morphology in female rats (100×, H&E). (**A**) Control group (C); (**B**) Control group with inflammatory cell infiltration (arrow); (**C**) Melatonin group (M); (**D**) Experimental oil diet group (E); (**E**) Melatonin + experimental oil diet group (ME). Arrow: inflammation. Abbreviations: C, control; M, melatonin; E, experimental oil diet; ME, melatonin + experimental oil diet.

After the 8-week intervention with the experimental oil diet and melatonin supplementation, gWATs were collected and stained with H&E. A histological evaluation under 100× magnification was performed to assess adipocyte hypertrophy, necrosis, inflammation, and the presence of brite/beige adipocytes, as shown in Figure 7. No evidence of brite/beige adipocytes, adipocyte hypertrophy, or necrosis was observed in any group. However, localized inflammatory cell infiltration was noted in the control group (Figure 7B). Nevertheless, the comparison of overall pathological scores revealed no significant variation between the groups.

### 3.11. Irisin and Irisin-Related Levels

**Figure 8:**
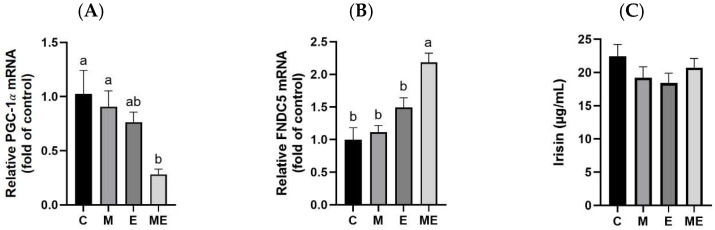
Impact of melatonin administration and the experimental oil diet on peroxisome proliferator-activated receptor gamma coactivator-1α (PGC-1α) and fibronectin type III domain-containing protein 5 (FNDC5) gene expression in muscle tissue, as well as serum irisin concentrations, in female rats. Shown are relative levels of (**A**) PGC-1α, (**B**) FNDC5, and (**C**) circulating irisin. Gene expression values were normalized to β-actin using the 2^−ΔΔCt^ approach, with the C group serving as the baseline for relative quantification. Results are presented as mean ± standard error of the mean from eight independent biological replicates. Statistical analysis employed one-way ANOVA followed by Tukey’s post hoc test, with significance set at *p* < 0.05. Different lowercase letters denote statistically significant differences between groups. Group abbreviations: C, control; M, melatonin; E, experimental oil diet; ME, melatonin + experimental oil diet.

After the 8-week intervention with the experimental oil diet and melatonin supple-mentation, serum irisin levels and mRNA expressions of PGC-1α and FNDC5 were evaluated, as shown in Figure 8. As illustrated in Figure 8A, the ME group exhibited markedly reduced PGC-1α mRNA levels compared with the C group (*p* = 0.005) and the M group (*p* = 0.02), and showed a lower value than the E group, although the difference was not statistically significant (*p* = 0.099). Conversely, FNDC5 mRNA levels were substantially elevated in the ME group when compared with C (*p* < 0.0001), M (*p* = 0.0001), and E (*p* = 0.013) groups (Figure 8B). Serum irisin concentrations did not differ significantly across experimental groups (Figure 8C).

## 4. Discussion

Using a female rat model, this study assessed the influence of melatonin administration and a tailored oil diet—formulated with a PUFA/SFA ratio of 5 and 60% MUFAs—on both lipid metabolism and body composition. A central finding of the present study is that the observed transcriptional changes in lipid metabolism-related genes were not accompanied by measurable physiological improvements at the whole-animal level. Despite modulation of selected mRNA markers, no significant differences were detected in body weight, adipose tissue mass, serum lipid profiles, or hepatic lipid content among groups. These results indicate that, under non-obese and physiologically normal conditions, transcriptional alterations alone were insufficient to elicit detectable metabolic benefits within the study duration.

The literature suggests that melatonin exerts a regulatory influence over circadian timing, energy regulation, and lipid metabolic activity [26,27]. It was also shown to enhance the lipolytic capacity in muscle tissues by activating the cyclic adenosine monophosphate (cAMP) and extracellular signal-regulated kinase 1/2 (ERK1/2) signaling pathways, thereby increasing the expression and activity of the HSL lipolytic gene, as well as promoting mitochondrial thermogenesis via elevated PGC-1α and CPT-1β activities [10,28,29,30]. Furthermore, the dietary FA composition is known to influence the activity of lipid-metabolizing enzymes and regulate pathways related to FA oxidation [16,31]. Specifically, diets with a high MUFA content and a high PUFA/SFA ratio are associated with improved adipose tissue function and a reduced risk of chronic inflammation [17,18,32,33].

Despite the absence of significant effects on BW, fat mass, or serum lipid concentrations, the 8-week regimen led to relevant shifts in the expression of genes regulating lipid metabolism within both hepatic tissue and WAT. Notably, in the ME group, hepatic mRNA expression levels of PPARα and ACO were significantly upregulated, suggesting that this combined intervention may enhance FA oxidation and mitochondrial metabolic function. These results are consistent with previous findings, which demonstrated that diets high in MUFAs and with a high PUFA/SFA ratio enhanced hepatic FA oxidation, supporting the role of the FA composition in modulating hepatic lipid metabolism via nu-clear receptors such as PPARα [17,18,19].

Furthermore, previous studies have reported that melatonin can enhance the expression of uncoupling protein 1 (UCP1), PGC-1α, and CPT-1, thereby promoting fatty acid oxidation and mitochondrial biogenesis through activation of the AMPK pathway [9,10,11,34,35]. In the present study, although the M group exhibited a higher caloric intake, this was not accompanied by significant changes in body weight, suggesting that melatonin may influence energy balance, but the precise mechanisms could not be determined from our data. Prior research has also shown that melatonin can activate AMPK, suppress ACC and SREBP-1c, reduce malonyl-CoA and FAS expression, and simultaneously upregulate CPT-1, PGC-1α, and PPARγ, favoring lipid oxidation over lipogenesis [35,36,37]. On the other hand, diets enriched in unsaturated fatty acids have similarly been reported to induce AMPK phosphorylation and inhibit lipogenic enzyme activities, though these effects may vary depending on sex and estrogen status [18,19]. Taken together, our findings at the transcriptional level suggest that melatonin and the experimental oil diet may act through distinct but potentially complementary pathways to modulate hepatic lipid metabolism, although these changes did not translate into measurable phenotypic outcomes in this study.

In WAT, PPARγ is abundantly expressed and plays an essential role in regulating lipid metabolism, including lipolysis and adipocyte differentiation [38,39]. Its activation can increase HSL expression, thereby promoting the hydrolysis of triglycerides in adipocytes and facilitating the release of free fatty acids and glycerol [19,40]. In the present study, HSL mRNA expression was significantly upregulated in the E group, suggesting that the dietary fatty acid composition may have influenced lipolytic activity. In contrast, PPARγ and LPL expression levels did not differ significantly among groups, which is consistent with the lack of marked histological differences in WAT. Although previous studies reported that melatonin supplementation suppressed PPARγ and LPL expression in adipose tissues of OVX rats, and that dietary fatty acid composition could upregulate PPARγ expression without affecting LPL [19,20], these effects were not evident in the current experimental setting. Moreover, no evidence of adipocyte hypertrophy or widespread inflammatory infiltration was observed across groups, apart from localized immune cell infiltration in the control group. Taken together, these results suggest that the transcriptional effects observed were modest and not accompanied by clear morphological or inflammatory changes, indicating that any potential protective effects of melatonin or the experimental oil diet on WAT metabolism may require longer interventions or different physiological contexts to become evident.

Prolonged physical exercise was shown to significantly enhance PGC-1α expression in skeletal muscles and cardiac tissues, subsequently promoting AMPK activation, phosphorylation of PGC-1α, and the upregulation of downstream FNDC5 expression [41,42]. FNDC5, a transmembrane protein, can be cleaved to produce irisin, a myokine involved in inducing thermogenesis and the browning of subcutaneous WAT. This process facilitates the generation of beige or brown adipocytes and has been associated with anti-obesity and glucose-regulating benefits [41,43]. In this study, after 8 weeks of a combined melatonin and experimental oil diet intervention, rats in the ME group exhibited significantly elevated FNDC5 mRNA expression in the liver, whereas PGC-1α expression was reduced, and no significant changes in circulating irisin levels were observed. Earlier research has shown that the FNDC5/irisin pathway is mainly triggered by physiological factors, including physical activity and exposure to cold, and facilitates the browning of adipose tissue and enhancement of thermogenesis through the AMPK–PGC-1α signaling cascade [42,44]. Melatonin was also shown to enhance expressions of thermogenic genes such as PGC-1α and UCP1 and to increase circulating irisin under specific conditions [9,20,45]. The paradoxical finding of increased FNDC5 expression accompanied by reduced PGC-1α and unchanged circulating irisin suggests that isolated transcriptional changes are insufficient to support activation of the FNDC5/irisin pathway or functional metabolic adaptation. This discordant regulation indicates that additional physiological stimuli—such as exercise, cold exposure, or an obesogenic challenge, which were absent in the present study—may be required to elicit systemic effects.

Overall, melatonin combined with a high-MUFA diet induced modest transcriptional modifications in lipid oxidation-related genes (PPARα, ACO, HSL) in the liver and adipose tissue, yet these were not accompanied by improvements in body weight, adiposity, or serum lipid profiles. These results indicate limited functional impact at the whole-animal level. Future studies employing longer interventions, physiologically relevant melatonin doses, and metabolic stress models are needed to clarify whether such dietary–hormonal interactions can provide meaningful benefits for systemic lipid metabolism.

## 5. Conclusions

In conclusion, an 8-week intervention with a high-MUFA diet and daily melatonin supplementation resulted in modest and non-uniform transcriptional changes in lipid metabolism-related genes in hepatic and adipose tissues of female rats under non-obese conditions, including alterations in genes associated with lipid oxidation. However, these molecular responses were not accompanied by measurable improvements in body weight, adiposity, or serum lipid profiles. Accordingly, the present findings should be interpreted as exploratory observations at the transcriptional level, rather than evidence of functional metabolic modulation or obesity prevention. Further studies employing metabolically challenged models (e.g., diet-induced obesity) and longer intervention periods are warranted to clarify the physiological relevance of these molecular responses.

## Figures and Tables

**Table 1:** Composition of the experimental diets (g/kg diet).

Ingredient	Control Diet	Experiment Diet
Casein	140	140
L-Cystine	1.8	1.8
Corn starch	460.7	460.7
Sucrose	100	100
Cellulose	50	50
Soybean oil	200	14
Canola oil	0	186
AIN-93 mineral mix	35	35
AIN-93 vitamin mix	10	10
Choline bitartrate	2.5	2.5
Total weight (g)	1000	1000
Total calories (kcal/kg diet)	4546.8	4546.8
Carbohydrate (% of total kcal)	49.3	49.3
Fat (% of total kcal)	39.6	39.6
Protein (% of total kcal)	11.1	11.1
Total SFAs	19.97	7.62
Total MUFAs	22.93	55.62
Total PUFAs	57.10	36.76
S/M/P proportion	1:1.15:2.85	1:7.3:4.82
P/S ratio	2.85	4.82

Abbreviations: SFAs, saturated fatty acids; MUFAs, monounsaturated fatty acids; PUFAs, polyunsaturated fatty acids; S/M/P, saturated/monounsaturated/polyunsaturated fatty acids; P/S, polyunsaturated/saturated fatty acids.

**Table 2:** List of primers sequences for the RT-qPCR.

	Forward Sequence (5′→3′)	Reverse Sequence (5′→3′)
ACC	GGAAGACCTGGTCAAGAAGAAAAT	CACCAGATCCTTATTATTGT
ACO	CTCCTCTGCCAACACCAACA	TAACGCTGGCTTCGAGTGAG
AMPK	ATGCCACTTTGCCTTCCGT	GCAGTTGCCTACCACCTCAT
CPT-1	CACGAAGCCCTCAAACAGATC	CCATTCTTGAACCGGATGAAC
FAS	AGCGGGAAAGTGTACCAGTG	GTAGCCGCAGCTCCTTGTAT
FNDC5	AGGACAACGAGCCCAATAAC	CATATCTTGCTTCGGAGGAGAC
HSL	CGCGGACCAGCTCTAAAGAA	ATGATGGCACCTCCCTTTGG
LPL	ATGGCACAGTGGCTGAAAGT	CCGGCTTTCACTCGGATCTT
PPARα	TCCTCTGGTTGTCCCCTTGA	TGTCAGTTCACAGGGAAGGC
PPARγ	GGGTGAAACTCTGGGAGATCCT	AGTGCTCATAGGCAGTGCATC
PGC-1α	TTCAGGAGCTGGATGGCTTG	GGGCAGCACACTCTATGTCA
SREBP-1c	AGGAGGCCATCTTGTTGCTT	GTTTTGACCCTTAGGGCAGC
β-actin	CACCAGTTCGCCATGGATGACGA	CCATCACACCCTGGTGCCTAGGGC

ACC, acetyl-CoA carboxylase; ACO, acyl-CoA oxidase; AMPK, 5′ adenosine monophosphate-activated protein kinase; CPT-1, carnitine palmitoyl transferase-1; FAS, fatty acid synthase; FNDC5, fibronectin type 3 domain-containing protein 5; HSL, hormone sensitive lipase; LPL, lipoprotein lipase; PPARα, peroxisome proliferator-activated receptor alpha; PPARγ, peroxisome proliferator-activated receptor gamma; PGC-1α, peroxisomal proliferator-activated receptor g coactivator-1α; SREBP-1c, sterol regulatory element-binding protein 1c.

**Table 3:** Influence of the experimental oil diet and melatonin administration on reproductive endocrine variables in female rats after 8-week intervention.

	C	M	E	ME
FSH (pg/mL)	7.52 ± 1.39	8.39 ± 0.82	8.37 ± 0.83	8.31 ± 0.98
Estradiol (pg/mL)	1580.41 ± 45.05	1679.06 ± 99.24	1542.55 ± 81.81	1573.41 ± 84.54
Uterus weight (g)	0.66 ± 0.11	0.71 ± 0.13	0.80 ± 0.07	0.78 ± 0.06
Relative uterus weight (g/100 g BW)	0.22 ± 0.03	0.24 ± 0.04	0.28 ± 0.02	0.27 ± 0.02

Serum female hormone concentrations and uterus weight after 8 weeks of the melatonin supplementation and experimental oil diet intervention. All values are presented as the mean ± standard error of the mean (*n* = 8). No statistically significant differences were identified among the experimental groups using one-way ANOVA with Tukey’s post hoc test. Group abbreviations: C, control; M, melatonin; E, experimental oil diet; ME, melatonin + experimental oil diet; FSH, follicle-stimulating hormone.

**Table 4:** Effect of the experimental oil diet and melatonin supplementation on body weight (BW) and food intake in female rats.

	C	M	E	ME
Initial BW (g)	224.19 ± 2.71	216.06 ± 3.24	219.69 ± 1.46	220.06 ± 2.17
BW after 8 weeks intervention (g)	300.38 ± 8.49	300.38 ± 7.11	286.38 ± 7.49	289.44 ± 10.58
Total weight gain (g)	76.19 ± 7.57	84.31 ± 7.11	66.69 ± 6.77	69.38 ± 9.42
Food intake (g/day/rat)	16.50 ± 0.51 ^ab^	16.72 ± 0.04 ^a^	15.48 ± 0.35 ^b^	16.59 ± 0.31 ^ab^
Dietary caloric intake (kcal/d)	75.03 ± 2.34 ^ab^	76.02 ± 0.2 ^a^	70.38 ± 1.59 ^b^	75.42 ± 1.39 ^ab^
Food efficiency (g BW gain/g diet)	8.17 ± 0.7	9.00 ± 0.76	7.65 ± 0.66	7.38 ± 0.88

Data are expressed as mean ± standard error of the mean (*n* = 8). Different superscript letters (a, b) within a row denote significant differences between groups (*p* < 0.05, one-way ANOVA with Tukey’s post hoc test). Group abbreviations: C, control; M, melatonin; E, experimental oil diet; ME, melatonin + experimental oil diet; BW, body weight. Food efficiency was calculated as (BW gain/food intake) × 100%.

**Table 5:** Effect of experimental oil diet and melatonin supplementation on liver and tissues weights in female rats.

	C	M	E	ME
Liver weight (g)	9.23 ± 0.62	8.53 ± 0.39	8.86 ± 0.34	8.79 ± 0.51
Gonadal adipose tissue weight (g)	6.41 ± 0.90	6.77 ± 0.50	6.66 ± 0.61	6.39 ± 0.64
Perirenal adipose tissue weight (g)	8.79 ± 1.67	9.83 ± 0.84	8.28 ± 1.19	9.92 ± 1.84
Quadriceps weight (g)	4.28 ± 0.08	4.28 ± 0.14	4.23 ± 0.12	4.06 ± 0.15
Gastrocnemius weight (g)	3.71 ± 0.05	3.50 ± 0.09	3.70 ± 0.10	3.38 ± 0.15
Relative liver weight (g)	3.07 ± 0.18	2.83 ± 0.07	3.09 ± 0.06	3.03 ± 0.12
Relative gonadal adipose tissue weight (g)	2.10 ± 0.27	2.25 ± 0.16	2.30 ± 0.16	2.20 ± 0.20
Relative perirenal adipose tissue weight (g)	2.84 ± 0.50	3.25 ± 0.23	2.84 ± 0.35	3.32 ± 0.49
Relative quadriceps weight (g)	1.44 ± 0.06	1.43 ± 0.05	1.48 ± 0.05	1.42 ± 0.09
Relative gastrocnemius weight (g)	1.24 ± 0.04	1.17 ± 0.03	1.30 ± 0.04	1.17 ± 0.05

All values are presented as the mean ± standard error of the mean (*n* = 8). No statistically significant differences were identified among the experimental groups using one-way ANOVA with Tukey’s post hoc test. Abbreviations: C, control group; M, melatonin group; E, experimental oil diet; ME, melatonin + experimental oil diet. Relative organ weights were expressed as g per 100 g of BW.

## Data Availability

Data supporting the results of this study are available from the corresponding author on reasonable request.
